# Practical Considerations and Successful Implementation of Vital Signs Monitoring. Comment on “Continuous Versus Intermittent Vital Signs Monitoring Using a Wearable, Wireless Patch in Patients Admitted to Surgical Wards: Pilot Cluster Randomized Controlled Trial”

**DOI:** 10.2196/14042

**Published:** 2021-03-11

**Authors:** Caoimhe Walsh, David Zargaran, Nikhil Patel, Amelia White, Foteini Stefania Koumpa, Ravina Tanna, Muhammad Arsalan Ashraf

**Affiliations:** 1 Epsom and St Helier University Hospitals NHS Trust Surrey United Kingdom; 2 Guy's and St Thomas' NHS Foundation Trust London United Kingdom; 3 Oxford University Hospitals NHS Foundation Trust Oxford United Kingdom; 4 Royal Free London NHS Foundation Trust London United Kingdom; 5 London North West University Healthcare NHS Trust London United Kingdom; 6 Luton and Dunstable University Hospital NHS Foundation Trust London United Kingdom

**Keywords:** general surgery, monitoring, observations, vital signs

We read with great interest the study by Downey et al [1], which examined the clinical utility and practicality of introducing a wearable, wireless patch device for continuous vital signs monitoring in surgical inpatients. As surgical trainees, we frequently rely on the recording of patients’ vital signs on observation charts and the use of early warning scores. These systems are in place as a safety mechanism to highlight acutely unwell patients and those at risk of clinical deterioration [2]. However, we are also all too familiar with the limitations posed by current methods of collecting and communicating this critical information, and there is a real need for further improvements in both of these areas [3]. 

Having a continuous monitoring device poses a number of potential benefits. First, by having many more data points clinicians can more accurately define long-term trends for each patient. Even more importantly, deterioration can be highlighted much sooner than currently permitted due to the 5-6–hour intervals between “observation rounds”—but will this result in information overload? It is easy to capture information, but this can be misleading, and overinterpretation of incidental or minor alterations in vital signs may lead to unnecessary additional investigations for a patient. Furthermore, although data are being collected continuously, nursing staff can only check this at discrete time points due to other jobs and ward responsibilities. Therefore, staff may nevertheless fail to recognize unwell patients at the earliest time point. This is evidenced by the fact that it still took an average of 626 minutes to initiate treatment for sepsis in the patient cohort with continuous monitoring, despite the UK national guidelines for the management of sepsis stating that antibiotics should be administered within 1 hour of the patient first being suspected to have sepsis [4].

We understand that the software initially overwhelmed nursing staff with false alerts, which highlights the important yet challenging balance to make between false alerts and detecting significant clinical changes. We would be interested to understand what parameters were used for the vital signs alerts, and whether these parameters were standardized across all patients or relative to the individual patient’s baseline vital signs. Furthermore, does the alert system indicate the severity of the trigger?

There is also the consideration of how practical it is for patients to wear such devices continuously around their chest. Anecdotally, we know that patients who are advised to wear TED (thromboembolism-deterrent) stockings as part of venous thromboembolism prophylaxis are often found to have removed them “temporarily” due to discomfort or to have a shower, with subsequent difficulty putting them back on. We can envisage similar factors affecting the wearing of these devices for vital signs monitoring; for example, they may be removed if they interfere with clinical examination of the chest, or again due to comfort or personal hygiene reasons. We already anticipate this happening as the study noted that 24% of patients did not wear the wireless patch for the whole length of their admission. This means that as well as providing training for nurses, doctors and indeed patients themselves would also need to be trained in how to position the devices on the chest to ensure they are placed back correctly if removed for whatever reason.

One solution to minimize the impact of wearing such devices would be to reserve them for use out of hours. Typically, this is when wards are minimally staffed and clinicians are individually responsible for a far larger number of patients, leading to a more significant need to highlight deteriorating patients [5]. During this time, patients are typically less active therefore there would be fewer false alarms, for example, from increased heart rate due to patients mobilizing, and with patients unlikely to be showering overnight, which would result in reduced chances for the need to remove or replace the device. Within normal working hours, nurses and doctors have a much greater presence in the ward; therefore, concerns regarding unwell patients are more likely to be raised in a timely manner. Thus, it may be that during the day, current methods of intermittently collecting vital signs data will continue to suffice; this would also reduce the interference of wearing the device with activities such as showering or being clinically examined.

We recommend that future studies control for the time of day when the clinical deterioration occurred. By analyzing separately the time to treat sepsis both in hours and out of hours for the continuously monitored and intermittently monitored groups, it would be possible to identify more objectively whether there is a particular time of day where continuous vital signs monitoring renders the greatest clinical benefit over intermittent monitoring.

This paper [1] has identified a sensible and considered solution to the issue of collecting and communicating vital signs data on surgical inpatients. Despite further work being required to streamline the implementation of this system into clinical use, we commend the authors on their innovative device. We hope this technology will soon help to improve clinical outcomes and look forward to seeing a study with a more significant population size, and thus greater power, to enable stronger conclusions to be drawn from the results.

## Abbreviations

TED: thromboembolism-deterrent
